# A Novel SIRT1 Activator Hydroxygenkwanin Alleviates Osteoporosis by Inhibiting Ferroptosis and Lactylation in Skeletal Stem/Progenitor Cells

**DOI:** 10.3390/antiox15050612

**Published:** 2026-05-12

**Authors:** Yu Zhai, Linhai Cao, Hao Li, Shengwen Cheng, Jiaying Wei, Xinhang Li, Wenjing Tang, Chen Zhao, Wei Huang, Minghan Liu

**Affiliations:** 1 Orthopaedic Research Laboratory, Department of Orthopaedic Surgery, The First Affiliated Hospital, Chongqing Medical University, Chongqing 400016, China; zhaiyu9501@tmmu.edu.cn (Y.Z.); 2023140147@stu.cqmu.edu.cn (S.C.); 2023130063@stu.cqmu.edu.cn (J.W.); 2023110205@stu.cqmu.edu.cn (X.L.); 2022140040@stu.cqmu.edu.cn (C.Z.); 2 Chongqing Municipal Health Commission Key Laboratory of Musculoskeletal Regeneration and Translational Medicine, Chongqing Medical University, Chongqing 400016, China; 3 Chongqing Municipal Health Commission Key Laboratory of Precise Orthopedics, Department of Orthopaedic Surgery, The Second Affiliated Hospital, Army Medical University, Chongqing 400038, China; hlc2392969071c@email.swu.edu.cn (L.C.); gklihao@tmmu.edu.cn (H.L.); tyche_93@tmmu.edu.cn (W.T.)

**Keywords:** osteoporosis, SIRT1, ferroptosis, lactylation, natural compound

## Abstract

Sirtuin 1 (SIRT1) is an important protein for maintaining cellular homeostasis, and targeting SIRT1 represents a promising strategy for alleviating osteoporosis. The discovery of highly potent and safe SIRT1 activators therefore holds significant translational value for clinical anti-osteoporosis therapies. In this study, we performed deep mining of high-throughput RNA-sequencing (RNA-seq) data from 576 young and aged skeletal stem/progenitor cells (SSPCs) and identified SIRT1 downregulation as a critical hallmark of SSPC ferroptosis during aging-related osteoporosis. In SIRT1 heterozygous deficiency (SIRT1^+/−^) mice, we found that SIRT1 deficiency triggered SSPC ferroptosis and induced premature osteoporosis. Computer-aided drug design (CADD) was employed to screen 9634 compounds targeting the SIRT1 active site, leading to the identification of the natural compound Hydroxygenkwanin (HGK) as a novel SIRT1 activator. HGK treatment effectively restored SIRT1 activity, suppressed ferroptosis in SSPCs in vitro, and ameliorated osteoporosis in vivo. Through transcriptomic analysis and lactylation profiling, we further found that HGK can activate SIRT1 and reverse the lactylation-mediated suppression of the enzymatic activities of SOD1 and PRDX1. This mechanism may underlie the ability of HGK to reduce SSPC ferroptosis and alleviate osteoporosis. Overall, our findings suggest that HGK possesses translational potential for the treatment of osteoporosis through SIRT1 activation.

## 1. Introduction

Osteoporosis is a systemic skeletal disorder characterized by low bone mass and microarchitectural deterioration of bone tissue, leading to increased bone fragility and susceptibility to fractures [1,2]. Epidemiologically, osteoporosis constitutes a major global public health burden associated with aging, with approximately 1 in 2 women and 1 in 4 men over the age of 50 affected [3]. Osteoporosis is a disease spectrum that begins with the silent loss of bone mass [4], progresses to the first fragility fracture (which may occur in the vertebrae, hip, wrist, humerus, or even ankle) [5], and may then lead to secondary or multiple fractures due to treatment gaps, accompanied by a series of issues such as deformities, pain, functional impairment, and surgical complications (e.g., internal fixation failure, nonunion) [6,7,8,9]. Additionally, osteoporosis often emerges as a complication of various chronic diseases (e.g., gastrointestinal disorders, rheumatism) or specific drug treatments (e.g., glucocorticoids) [10,11,12]. Current therapeutic strategies for osteoporosis primarily include anti-resorptive agents (e.g., bisphosphonates) and anabolic agents (e.g., teriparatide) [13], which can alleviate bone loss to a certain extent but are associated with side effects such as gastrointestinal disorders and osteonecrosis of the jaw [14,15]. Moreover, these drugs fail to fully reverse the impairment of bone microarchitecture and regenerative capacity [16], highlighting the urgent need to explore novel therapeutic targets and mechanisms underlying osteoporosis progression.

Skeletal Stem/Progenitor Cells (SSPCs) are a subset of multipotent stem cells that possess self-renewal capacity and lineage specificity, enabling them to differentiate into osteoblasts and chondrocytes [17,18]. Compared with bone marrow mesenchymal stem cells (BMSCs), SSPCs possess stronger bone-forming potential, more precise lineage commitment, and higher sensitivity to skeletal microenvironmental cues, making them the core functional cells responsible for skeletal development, maintenance of homeostasis, and bone repair [19,20]. Accumulating evidence has demonstrated that SSPCs play a pivotal role in embryonic skeletal morphogenesis and postnatal bone regeneration [21]. The dysfunction of SSPCs is closely associated with impaired bone healing and age-related bone loss [22,23]. However, the molecular mechanisms underlying SSPC dysfunction in bone regeneration, especially in the context of osteoporosis, remain poorly elucidated, which limits the development of SSPC-based therapeutic strategies for bone degenerative diseases.

The Sirtuin (SIRT) family comprises NAD^+^-dependent deacylases that regulate various cellular processes, including maintaining cellular metabolic homeostasis, regulating oxidative stress responses, and protecting cells from ferroptosis, by modifying both histone and non-histone proteins [24,25]. Among them, Sirtuin 1 (SIRT1) is the most extensively studied member, characterized by its broad substrate specificity and critical role in maintaining cellular homeostasis [26,27]. Emerging studies have established a connection between SIRT1 and various orthopedic degenerative diseases [28]. Specifically, SIRT1 has been reported to regulate osteoblast differentiation and osteoclast activation during osteoporosis [29,30]. In addition, SIRT1 activation has been shown to alleviate osteoarthritis and intervertebral disk degeneration [31,32]. Although SIRT1 activation represents a promising strategy to maintain cellular homeostasis of SSPCs and thereby treat osteoporosis, safe and highly potent SIRT1 activators suitable for clinical therapy are currently lacking.

In the present study, by mining large-cohort high-throughput RNA-sequencing (RNA-seq) data, we identified that decreased SIRT1 expression in SSPCs correlated with the progression of osteoporosis. Then, we assessed the impact of SIRT1 deficiency on osteoporosis development and SSPCs ferroptosis. In addition, we integrated libraries of repurposed compounds, dietary bioactive compounds, and FDA-approved compounds for high-throughput virtual screening and further investigated the therapeutic efficacy of Hydroxygenkwanin (HGK, Luteolin-7-methyl ether), a candidate SIRT1 activator, against SIRT1 deficiency-induced osteoporosis. Furthermore, we characterized the downstream molecular signaling of HGK in alleviating osteoporosis. Collectively, our findings provide a robust theoretical foundation for targeted activation of SIRT1 by HGK as a promising therapeutic strategy for osteoporosis.

## 2. Materials and Methods

### 2.1. High-Throughput RNA-Seq and Bioinformatics Analysis

RNA-seq Data comprising 576 SSPC samples (288 from young mice and 288 aged mice) were retrieved from the Gene Expression Omnibus (GEO) database under accession number GSE161946. Following data normalization, differential expression analysis was performed using the DESeq2 package in R (Version 1.50.2), where differentially expressed genes (DEGs) were identified based on the criteria of |log_2FoldChange| ≥ 1 and an adjusted *p*-value < 0.05. The expression patterns of significant DEGs were visualized using hierarchical clustering heatmaps and volcano plots. To further elucidate the underlying biological mechanisms, Gene Ontology (GO) and Kyoto Encyclopedia of Genes and Genomes (KEGG) enrichment analyses were conducted using the clusterProfiler package (Version 4.4.4), while Gene Set Enrichment Analysis (GSEA) was employed to specifically evaluate the enrichment status of ferroptosis-related signaling signatures in aged SSPCs. Finally, to screen for key ferroptosis regulators involved in osteoporosis, known ferroptosis driver and suppressor genes were retrieved from the FerrDb database (Version V3) and intersected with the identified DEGs to pinpoint candidate molecules, specifically focusing on the expression profiles of Sirtuin family members.

### 2.2. Generation of SIRT1 Heterozygous Deficiency (SIRT1^+/−^) Mice

SIRT1^+/−^ mice were generated on a C57BL/6J background via CRISPR/Cas9 [33]. sgRNA targeting the Exon 2-3 of SIRT1 (GenBank Accession No.: NM_019812.3, sequence: A1: CAGGTAGATTGCTACTTGAA-AGG, A2: CATGTGGATGCTAGCAAACC-TGG) was synthesized by Thermo Fisher Scientific. Cas9 mRNA was in vitro transcribed using the mMESSAGE mMACHINE T7 Ultra Kit (AM1345, Thermo Fisher Scientific, Waltham, MA, USA). sgRNA (50 ng/μL) and Cas9 mRNA (100 ng/μL) were microinjected into C57BL/6J zygotes, which were then cultured and transplanted into pseudopregnant ICR females. F0 mice were genotyped by PCR and Sanger sequencing. Stable SIRT1^+/−^ mice (F1 generation) were obtained by backcrossing F0 mice with WT C57BL/6J mice. Wild-type Littermates SIRT1^+/+^ mice (SIRT1^WT^) were set as the control. 2-month-old WT C57BL/6J mice, 24-month-old WT C56BL/6J mice were purchased from the animal center of Army Medical University. All mice were housed in SPF conditions (22 ± 2 °C, 50 ± 10% humidity, 12 h light/dark cycle) with ad libitum access to standard chow and sterile water. All animal experiments were approved by the Laboratory Animal Welfare and Ethics Committee of Army Medical University (AMUWEC20255465).

### 2.3. H&E Staining and Masson Staining

Paraffin-embedded 5 μm sections of femoral tissue from SIRT1^+/−^ and SIRT1^WT^ mice were stained using commercial hematoxylin/eosin (H&E) (G1120, Solarbio, Beijing, China) and Masson’s Trichrome kits (G1346-8, Solarbio, Beijing, China) per manufacturers’ protocols and previously described [34]. Briefly, sections were deparaffinized, rehydrated, stained with hematoxylin/eosin or ponceau-fuchsin/aniline blue, then dehydrated and mounted. Stained sections were imaged via an Olympus optical microscope (Olympus, Tokyo, Japan). The histological Region of Interest (ROI) selection and analysis of the femur in this study was based on standardized sampling using fixed anatomical landmarks. In accordance with the ASBMR principles for bone histomorphometry and rodent bone histomorphometry [35,36], H&E and Masson staining were analyzed in the trabecular region of the distal femoral metaphysis in mice, and comparisons were performed on anatomically matched sections. The ROI was defined within the secondary spongiosa rather than the primary spongiosa adjacent to the growth plate. The region within approximately 0.25 mm below the growth plate was excluded, and the region within approximately 0.25 mm from the endocortical bone surface was also excluded, in order to minimize the influence of the primary spongiosa and endocortical remodeling on quantitative analysis. The analysis area covered a standardized trabecular region of the distal femoral metaphysis, while cortical bone, the epiphyseal region, and sectioning artifacts were excluded. All specimens were analyzed using the same magnification and identical ROI selection criteria. H&E staining evaluated tissue morphology, while Masson staining assessed overall collagen deposition/bone matrix collagen in the trabecular region.

### 2.4. Immunofluorescence Staining for Osteocalcin (OCN)

Immunofluorescence Staining was performed as previously described [37]. 5 μm-thick paraffin-embedded sections of femoral tissue from SIRT1^+/−^ and SIRT1^WT^ mice were sequentially deparaffinized in xylene and rehydrated through a graded ethanol series, followed by antigen retrieval in citrate buffer (pH 6.0) using a heat-induced epitope retrieval protocol. Non-specific antibody binding was blocked by incubating sections in 5% bovine serum albumin (BSA) for 30 min at 37 °C. Sections were then incubated overnight at 4 °C with a rabbit monoclonal primary antibody targeting OCN (dilution 1:100, GB11233, Servicebio, Wuhan, China). After three washes with phosphate-buffered saline containing 0.1% Tween-20, sections were incubated with a fluorescently conjugated goat anti-rabbit secondary antibody (dilution 1:200, SA00013-2, Proteintech, Rosemont, IL, USA) for 1 h at 37 °C in the dark. Nuclei were counterstained with Hoechst 33,342 (Servicebio, Wuhan, China) at a concentration of 1 μg/mL for 5 min. Fluorescent images were captured using a confocal laser scanning microscope (Olympus, Tokyo, Japan). OCN-positive cells were identified by green fluorescent signals, while nuclei were visualized as blue signals.

### 2.5. Isolation, Identification, and Culture of SSPCs

Primary SSPCs were isolated from the femoral tissue of 2-month-old or 6-month-old SIRT1^WT^ and SIRT1^+/−^ mice. SIRT1^WT^ and SIRT1^+/−^ mice via density gradient centrifugation combined with flow cytometry sorting. Tissue was minced, digested with 0.2% collagenase I (Sigma-Aldrich, Louis, MO, USA) at 37 °C for 30 min, filtered (70 μm), and centrifuged. Cells were layered onto 1.073 g/mL Percoll (GE Healthcare, Chicago, IL, USA), centrifuged, and the intermediate layer was collected. The isolation of SSPCs (CD45−, TER119−, TIE2−, CD51+, CD200+, 6C3−, CD105−) was performed as previously described [38,39]. SSPCs were cultured in α-MEM (Gibco, Grand Island, NY, USA) supplemented with 10% FBS, 100 U/mL penicillin, and 100 μg/mL streptomycin (Solarbio, Beijing, China) at 37 °C/5% CO_2_.

### 2.6. FerroOrange Staining

SSPCs were seeded on confocal coverslips. Cells were rinsed with pre-warmed PBS, incubated with 1 μM FerroOrange (G1727, Servicebio, Wuhan, China) in serum-free medium at 37 °C and 5% CO_2_ for 30 min, and washed three times with PBS. The nucleus was counterstained with Hoechst (1 μg/mL) for 5 min. Imaging was performed on a confocal microscope (FerroOrange: Ex/Em 555/580–630 nm; Hochest: 405/440–480 nm) with standardized parameters. Average fluorescence intensity per cell was quantified via ImageJ (Version 1.54).

### 2.7. JC-1 Staining

JC-1 staining was used to evaluate mitochondrial membrane potential (MMP) via the ratio of J-aggregate (red) to monomer (green) fluorescence. SSPCs were seeded on coverslips, rinsed with PBS, and incubated with 5 μM JC-1 (C2005, Beyotime, Shanghai, China) in serum-free α-MEM at 37 °C/5% CO_2_ for 20 min. After 3× PBS washes, nuclei were stained with Hoechst (1 μg/mL). Imaging was performed on a confocal microscope (JC-1 aggregates: 555/580–630 nm; monomers: 488/520–550 nm). The red-to-green fluorescence ratio (ΔΨₘ indicator) was quantified via ImageJ.

### 2.8. BODIPY Staining

The lipid peroxidation reporter BODIPY where red-to-green fluorescence shift indicates oxidized lipids were used to evaluate ferroptosis level. SSPCs were seeded on coverslips, rinsed with PBS, and incubated with 5 μM BODIPY (G1733, Servicebio, Wuhan, China) in serum-free α-MEM at 37 °C/5% CO_2_ for 30 min. After 3× PBS washes, imaging was performed on a confocal microscope (reduced BODIPY: 581/591 nm; oxidized: 488/515 nm) with standardized parameters. The green-to-red ratio was quantified via ImageJ.

### 2.9. Micro-CT Analysis

Micro-CT was performed using a Bruker Skyscan system (Bruker microCT N.V., Kontich, Belgium) to assess the degree of osteoporosis in ex vivo femur specimens. Femur samples were vertically fixed in the scanning tube, and scanning parameters were set as voltage of 70 kV, tube current of 114 μA, integration time of 300 ms, and isotropic resolution of 6 μm. After scanning, image reconstruction and analysis were conducted using the associated workstation software CTAn (Version 1.18). Volume of Interest (VOI) selection for micro-CT analysis was standardized using the distal femoral growth plate as an anatomical reference according to previous studies [40,41]. Trabecular measurements were taken 0.78 mm from the growth plate reference and extended for 2.5 mm along the trabecular structure. This standardized approach was used to ensure that the same metaphyseal trabecular region was analyzed in all specimens and to minimize the influence of the primary spongiosa adjacent to the growth plate. In addition, the VOI was confined to the trabecular compartment of the distal femoral metaphysis, while non-trabecular regions, including cortical bone and non-target anatomical areas, were excluded from the analysis. All samples were processed and analyzed using the same anatomical landmark, offset distance, VOI length, thresholding strategy, and analysis workflow, thereby improving consistency and reproducibility across groups, and the following bone morphometric parameters including bone volume fraction (Bone Volume/Total Volume, BV/TV) and bone mineral density (BMD) were calculated. All parameter measurements were performed in accordance with the guidelines of the American Society for Bone and Mineral Research (ASBMR).

### 2.10. Western Blot of Pan-Lactylation and Lactylated Proteins Levels

Cells were lysed with RIPA buffer and protein concentration was quantified by the BCA assay. Equal amounts of protein were separated by SDS-PAGE, transferred to PVDF membranes, blocked, and incubated with pan-Lactylation primary antibody (PTM-1401RM, PTM BIO, Hangzhou, China) at 4 °C overnight, followed by HRP-conjugated secondary antibody. Signals were detected by ECL chemiluminescence (RM02867, Abclonal, Wuhan, China). Band intensities were quantified via ImageJ (normalized to total protein). For immunoprecipitation (IP) assays, protein A magnetic beads were pre-conjugated with either IgG (as a negative control) or antibodies including Superoxide Dismutase 1 (SOD1) (10269-1-AP, Proteintech, Rosemont, IL, USA), Peroxiredoxin 1 (PRDX1) (15816-1-AP, Proteintech, Rosemont, IL, USA), PEX14 (10594-1-AP, Proteintech, Rosemont, IL, USA), ACSL3 (20710-1-AP, Proteintech, Rosemont, IL, USA), and SOD2 (24127-1-AP, Proteintech, Rosemont, IL, USA). Subsequently, lysate proteins were incubated with either IgG- or antibodies-conjugated magnetic beads for IP. Proteins from both input and IP fractions were denatured using SDS-PAGE loading buffer (Beyotime, Shanghai, China). The immunoprecipitates were separated by SDS-PAGE, and the lactylation levels of the target proteins were detected by Western blotting using the Pan-Lactylation antibody as described above.

### 2.11. Quantitative Real-Time PCR (RT-qPCR)

Total RNA was extracted from cultured SSPCs using the TRIzol reagent (Invitrogen, Waltham, MA, USA) and quantified via a NanoDrop 2000 spectrophotometer (Thermo Fisher Scientific, Waltham, MA, USA). Subsequently, 1 μg of total RNA was reverse-transcribed into cDNA using the PrimeScript™ RT Reagent Kit with gDNA Eraser (Takara, Kusatsu, Shiga, Japan) to eliminate genomic DNA contamination. RT-qPCR was conducted on a LightCycler 480 II System (Roche, Basel, Switzerland) using TB Green^®^ Premix Ex Taq™ II (Takara, Kusatsu, Shiga, Japan) in a 10 μL reaction volume. The amplification protocol consisted of an initial denaturation at 95 °C for 30 s, followed by 40 cycles of denaturation at 95 °C for 5 s and annealing/extension at 60 °C for 30 s, ending with a melting curve analysis to verify specificity. The relative mRNA expression levels of Sirt1, Sirt2, Sirt3, and Sirt6 were normalized to the internal control Gapdh and calculated using the 2^−△△CT^ method.

### 2.12. RNA-Seq of SSCs from HGK-Treated SIRT1^+/−^ Mice

Total RNA was isolated from SSPCs in vehicle- or HGK-treated SIRT1^+/−^ mice, strictly following the manufacturer’s recommended protocol. RNA-seq services were provided by Majorbio Co., Ltd. (Shanghai, China), while RNA integrity was assessed using a NanoDrop 2000 spectrophotometer. For differential gene expression analysis, data filtration was implemented with the R package DESeq2, setting the cutoff criteria as |log2FC| > 1 and *p* value < 0.05. Sample quality parameters and raw read counts were loaded into the R environment for subsequent bioinformatic processing. Raw sequencing datasets were subjected to quality control checks, inter-group differential comparison, as well as the generation of gene expression heatmaps and volcano plots, all via R-based computational workflows. GO and KEGG pathway enrichment analyses and GSEA were carried out using the clusterProfiler package (Version 4.4.4) in R and Majorbio Cloud Platform, with the analysis based on predefined pathway gene set libraries. The RNA-sequencing data have been deposited in the NCBI BioProject database under accession number PRJNA1464077.

### 2.13. Measurement of PRDX1 and SOD1 Enzymatic Activities

For the detection of SOD1 (Cu/Zn-SOD) enzymatic activities, a Superoxide Dismutase Assay Kit (706002, Cayman Chemical, Ann Arbor, MI, USA) was employed. Briefly, cell lysates were centrifuged at 10,000× *g* to isolate the cytosolic fraction, thereby excluding interference from mitochondrial SOD2. The assay utilized a xanthine oxidase system to generate superoxide anions, and cytosolic SOD1 activity was calculated by measuring the inhibition of formazan formation at 450 nm using a microplate reader. For the assessment of PRDX1 activity, Total intracellular PRDX peroxidase activity was assessed using a Trx-TrxR-NADPH coupled assay [42]. Briefly, SSPC lysates (100 µg protein) were added to a 200 μL reaction mixture containing 50 mM HEPES-NaOH (pH 7.0), 200 µM NADPH, 3 µM yeast Trx1, and 1.5 µM yeast TrxR1. After initiating the reaction with 50 µM H_2_O_2_ at 30 °C, NADPH oxidation was monitored at 340 nm for 15 min (20 s intervals). The specific activity (μmol/min/mg protein) was calculated based on the initial linear rate (first 5 min) after subtracting the background oxidation rate measured in Trx/TrxR-free controls.

### 2.14. High-Throughput Computer-Aided Drug Design (CADD)-Based Virtual Screening

Structure-based virtual screening targeting the active site of SIRT1 was performed using Schrödinger Maestro version 11.4. Briefly, the X-ray crystal structure of SIRT1 (5BTR) was retrieved from the RCSB Protein Data Bank and refined using the Protein Preparation Wizard module integrated in Schrödinger to optimize hydrogen bonding networks and eliminate structural defects. The screening compound library was constructed by integrating three distinct compound collections from TargetMol Chemicals, Inc. (Boston, MA, USA), including Repurposing Compounds Library (SC1300), Dietary Bioactive Compound Library (L6300), and FDA-Approved Compounds Library (L1010). Duplicate entries across the integrated libraries were removed to yield an initial set of 9634 unique compounds. Ligand preparation was subsequently conducted to generate relevant protonation states and stereoisomers of these compounds, followed by energy optimization of the entire compound library using the LigPrep module to ensure structural stability for docking analysis, resulting in a final screening library with 22,759 entries. Compounds with a docking score < −5 were selected from the XP docking results, and Lipinski’s Rule of Five was applied to this subset to assess drug-likeness, ultimately yielding 651 qualified compounds. From these candidates, the top 10 compounds with the highest absolute docking scores with the SIRT1 active pocket harboring the GLU230 residue were chosen for in-depth analysis. 2D and 3D visualizations of compound-protein binding modes were generated using PyMOL software (Version 3.1.8).

### 2.15. In Vitro Verification of Candidate SIRT1 Activators

To validate the biological efficacy of the top 10 candidate compounds identified via virtual screening, SIRT1^+/−^ mice-derived SSPCs were seeded into 96-well plates. The 10 candidate compounds were purchased from MedChemExpress Co., Ltd. (Monmouth Junction, NJ, USA). Compounds were dissolved in DMSO and added to the culture medium at a final concentration of 10 μM for 3 days. SIRT1^WT^ mice-derived SSPCs were used as the reference to normalize SIRT1 activity. SIRT1^+/−^ mice-derived SSPCs treated with an equivalent volume of DMSO served as vehicle control. SIRT1 enzymatic activity was subsequently quantified using a commercial Fluorometric SIRT1 Activity Assay Kit (ab156065, Abcam, Cambridge, UK) according to the manufacturer’s protocol. Relative SIRT1 activity was normalized to the protein concentration of each sample and expressed as a fold change relative to the SIRT1^WT^ group.

### 2.16. HGK Treatment In Vitro and Vivo

1 μM or 10 μM HGK were diluted in DMSO and added to the medium for cultured SSPCs from 6-month old SIRT1^+/−^ mice for 3 days, followed by FerroOrange staining, JC-1 staining, and BODIPY Staining in vitro. For in vivo evaluation, 2-month-old SIRT1^+/−^ mice were randomly divided into a vehicle-treated group, a 1 mg/d group, and a 10 mg/d group. HGK was administered orally for 4 months using a voluntary gelatin ingestion method [43]. Briefly, an 8% gelatin stock solution containing 2% sucralose was prepared and stored at −20 °C. HGK was dissolved in glycerol and mixed with the gelatin stock and strawberry flavoring in a ratio of 450:1300:150 (*v*/*v*/*v*). During the HGK treatment period, mice were individually housed for 15 min daily to consume the gelatin containing either 1 mg/d or 10 mg/d of HGK. Complete consumption was visually verified to ensure dose accuracy.

### 2.17. Microscale Thermophoresis (MST) Test Detecting the Binding of HGK with SIRT1

The binding interaction between HGK and SIRT1 was analyzed by MST. Recombinant SIRT1 protein (Yunclone Biotechnology, Wuhan, China) was incubated with NT-647-NHS fluorescent dye (Cat. #A20006, Thermo Fisher Scientific, USA) in the dark for 30 min. HGK was tested at 16 serial concentrations. The reaction system was set in a buffer containing 50 mM HEPES (pH 7.4) and 0.05% Tween 20. MST detection was performed using a Monolith NT.115 instrument (NanoTemper Technologies, Munich, Germany). All experiments were carried out in triplicate, and data analysis was performed using the dedicated Monolith software (Version 2.3).

### 2.18. In Vivo Biosafety Evaluation

To assess potential systemic toxicity, the body weight of each mouse was recorded monthly. Major organs (heart, liver, spleen, lung, and kidneys) were dissected and weighed to calculate organ coefficients (organ weight/body weight × 100%). Peripheral blood was collected for serum biochemical analysis of liver function markers (ALT and AST) and for hematological profiling (including WBC, RBC, HGB, and PLT counts) using standard automated analyzers according to the manufacturers’ protocols.

### 2.19. Statistical Analysis

Statistical analyses were conducted utilizing GraphPad Prism version 9.0. Data are expressed as the means ± standard deviation (SD). All continuous numerical variables were tested for normal distribution via kurtosis and skewness methods. Each experimental result was calculated from at least three biological replicates with technical triplicates. Comparisons between two groups were analyzed using an unpaired Student’s *t*-test. For comparisons involving three or more groups, one-way analysis of variance (ANOVA) followed by Bonferroni’s post hoc test was performed. *p* < 0.05 was considered statistically significant. Researchers were blinded to the evaluation of experimental outcomes and the analysis of raw data.

## 3. Results

### 3.1. RNA-Seq Analysis Comparing SSPCs from Young Mice and Aged Mice

**Figure 1:**
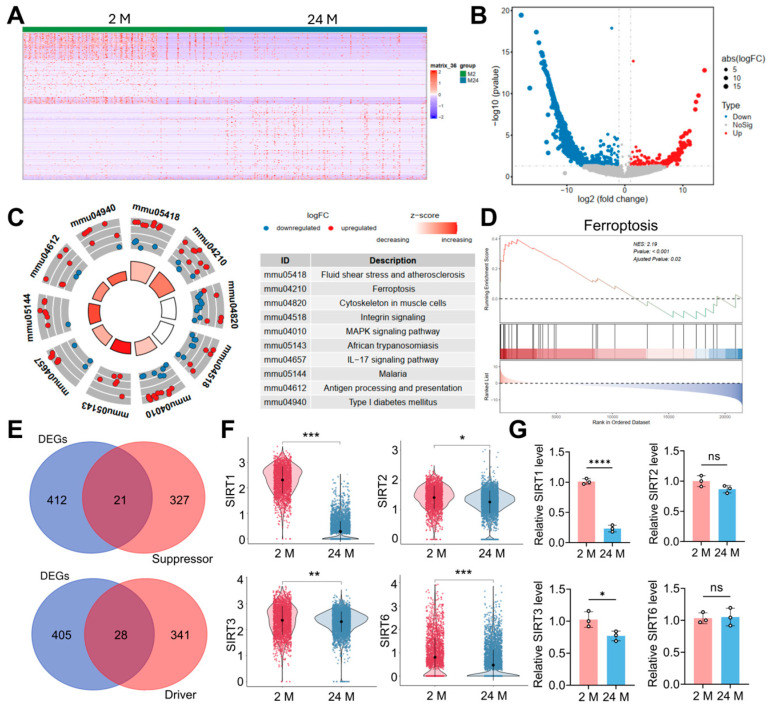
Transcriptomic Profiling Reveals Ferroptosis Activation and Sirtuin 1 (SIRT1) Downregulation in Aged Mice-Derived skeletal stem/progenitor cells (SSPCs). (**A**) Hierarchical clustering heatmap of differentially expressed genes (DEGs) in SSPCs isolated from 2-month-old (2 M) and 24-month-old (24 M) mice. Red means significantly upregulated genes, Blue means significantly downregulated genes. (**B**) Volcano plot visualizing the DEGs between the 2 M and 24 M groups. Red dots indicate significantly upregulated genes, blue dots indicate significantly downregulated genes, and gray dots represent non-significant genes. (**C**) Circos plot illustrating the Kyoto Encyclopedia of Genes and Genomes (KEGG) pathway enrichment analysis of the identified DEGs with key enriched pathways listed in the table. (**D**) Gene Set Enrichment Analysis (GSEA) plot showing the significant enrichment of the ferroptosis pathway in the 24 M group compared to the 2 M group. (**E**) Venn diagrams showing the intersection of DEGs (blue) with known ferroptosis suppressor genes (red) and driver genes (red). (**F**) Violin plots showing the relative mRNA expression levels of *SIRT1*, *SIRT2*, *SIRT3*, and *SIRT6* derived from the High-Throughput RNA-sequencing (RNA-seq) dataset. Each dot means a sample. (**G**) Quantitative Real-Time PCR (RT-qPCR) validation of the relative mRNA expression levels of *SIRT1*, *SIRT2*, *SIRT3*, and *SIRT6* in the 2 M and 24 M SSPCs. Each cycle means a sample. Data are presented as mean ± SD. Statistical significance was calculated with Student’s *t*-test (* *p* < 0.05; ** *p* < 0.01; *** *p* < 0.001; **** *p* < 0.0001; ns stands for no significant change).

We analyzed Smart-seq datasets from 288 young SSPCs (isolated from 2-month-old mice) and 288 aged SSPCs (isolated from 24-month-old mice). After filtering for low-expression genes, differential expression analysis was performed with criteria of |log_2FC| ≥ 1 and FDR < 0.05. This identified 433 differentially expressed genes (DEGs), including 225 upregulated and 208 downregulated genes, as visualized in the hierarchical clustering heatmap and volcano plot (Figure 1A,B). To characterize the biological functions of these DEGs, KEGG enrichment analyses were conducted. Notably, the DEGs were significantly enriched in ferroptosis-related pathways (Figure 1C). Consistent with these findings, GSEA demonstrated a significant enrichment of ferroptosis-related gene sets in the aged SSPC group (Figure 1D). To further pinpoint specific ferroptosis regulators, we intersected the identified DEGs with known ferroptosis suppressor and driver genes retrieved from the FerrDb database (Figure 1E). Among the ferroptosis suppressors, multiple members of the SIRT family, including *SIRT1*, *SIRT2*, *SIRT3*, and *SIRT6*, were found to be downregulated in aged SSPCs (Figure 1F). We isolated SSPCs from the femoral tissue of 2-month-old WT C56BL/6J mice, 24-month-old WT C56BL/6J mice, and subsequent RT-qPCR validation confirmed these findings and revealed that *SIRT1* exhibited the most profound downregulation among the SIRT family members in aged SSPCs compared to young controls (Figure 1G). Collectively, these transcriptomic analyses highlight a potential link between SIRT1 deficiency and SSPC ferroptosis during aging-related osteoporosis.

### 3.2. SIRT1 Deficiency Induces Premature Osteoporosis in Mice

**Figure 2:**
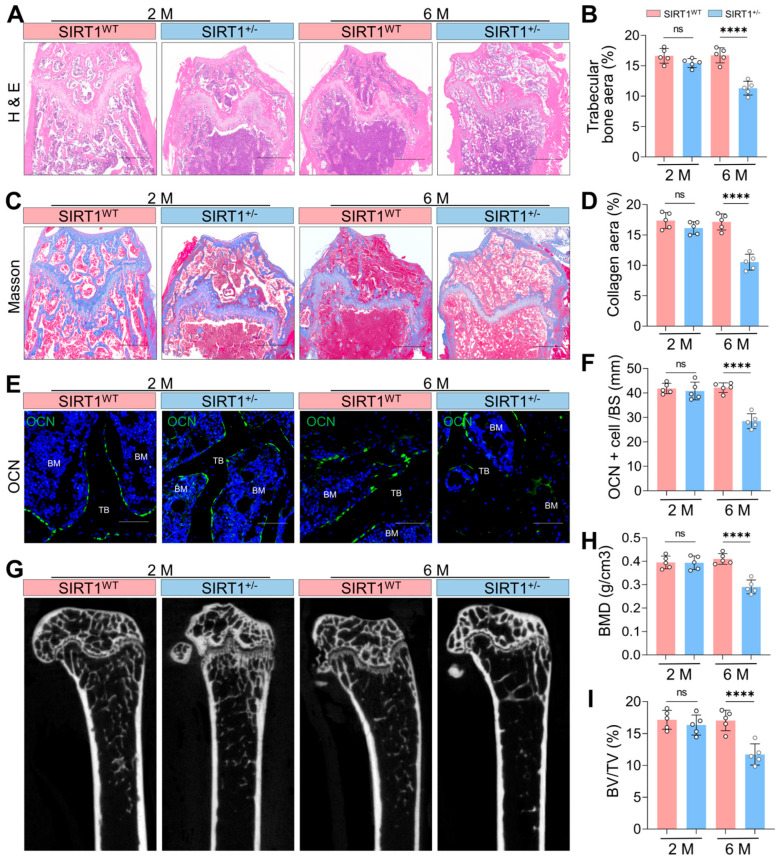
SIRT1 Deficiency Leads to Premature Osteoporosis in Mice. (**A**) Representative H&E staining images of the femoral bone in SIRT1^WT^ and SIRT1 heterozygous deficiency (SIRT1^+/−^) mice aged 2 and 6 months. Scale bar = 400 μm. (**B**) Quantification of femoral trabecular bone in SIRT1^WT^ and SIRT1^+/−^ mice aged 2 and 6 months. *n* = 5. (**C**) Representative Masson staining images of the femoral bone in SIRT1^WT^ and SIRT1^+/−^ mice aged 2 and 6 months. Scale bar = 400 μm. (**D**) Quantification of collagen areas in SIRT1^WT^ and SIRT1^+/−^ mice aged 2 and 6 months. *n* = 5. (**E**) Immunofluorescence staining images of Osteocalcin (OCN) to assess osteoblast functional status in SIRT1^WT^ and SIRT1^+/−^ mice aged 2 and 6 months. BM indicates bone marrow, TB indicates trabecular bone. Scale bar = 100 μm. (**F**) Quantification of the numbers/BS of OCN-positive mature osteoblasts in SIRT1^WT^ and SIRT1^+/−^ mice aged 2 and 6 months. *n* = 5. Data are presented as mean ± SD. (**G**) Representative coronal section micro-CT images of the femoral bone in SIRT1^WT^ and SIRT1^+/−^ mice aged 2 and 6 months. (**H**,**I**) Quantification of the bone mineral density (BMD) and Bone Volume/Total Volume (BV/TV) of the femoral bone in SIRT1^WT^ and SIRT1^+/−^ mice aged 2 and 6 months. Data are presented as mean ± SD. Each cycle means a sample. Statistical significance was calculated with one-way ANOVA for multiple-group comparisons (**** *p* < 0.0001; ns stands for no significant change).

To elucidate the functional role of SIRT1 in SSPCs, we employed transgenic animal models for follow-up investigations. Given that global knockout of the SIRT1 gene leads to embryonic lethality [44], we generated SIRT1^+/−^ mice for subsequent phenotypic characterization. We profiled the skeletal phenotypes of these mice at 2 and 6 months of age. H&E staining revealed no significant difference in trabecular bone area between SIRT1^WT^ and SIRT1^+/−^ mice at 2 months. However, by 6 months, SIRT1^+/−^ mice displayed a dramatic reduction in trabecular bone area relative to age-matched SIRT1^WT^ controls (Figure 2A,B). Consistently, Masson staining demonstrated that 6-month-old SIRT1^+/−^ mice exhibited a pronounced reduction in collagen matrix deposition (Figure 2C,D). In addition, SIRT1^+/−^ mice showed a substantial decrease in the number of OCN-positive mature osteoblasts (Figure 2E,F). Using Micro-CT analysis, we found that there is no significant difference in BMD and BV/TV value between SIRT1^WT^ and SIRT1^+/−^ mice at 2 months, while the BMD and BV/TV value were downregulated in SIRT1^+/−^ mice compared with SIRT1^WT^ at 6 months (Figure 2G–I). Collectively, these phenotypic analyses demonstrate that SIRT1 deficiency leads to impairment of bone homeostasis, characterized by premature osteoporosis in mice.

### 3.3. SIRT1 Deficiency Triggers Ferroptosis in SSPCs

**Figure 3:**
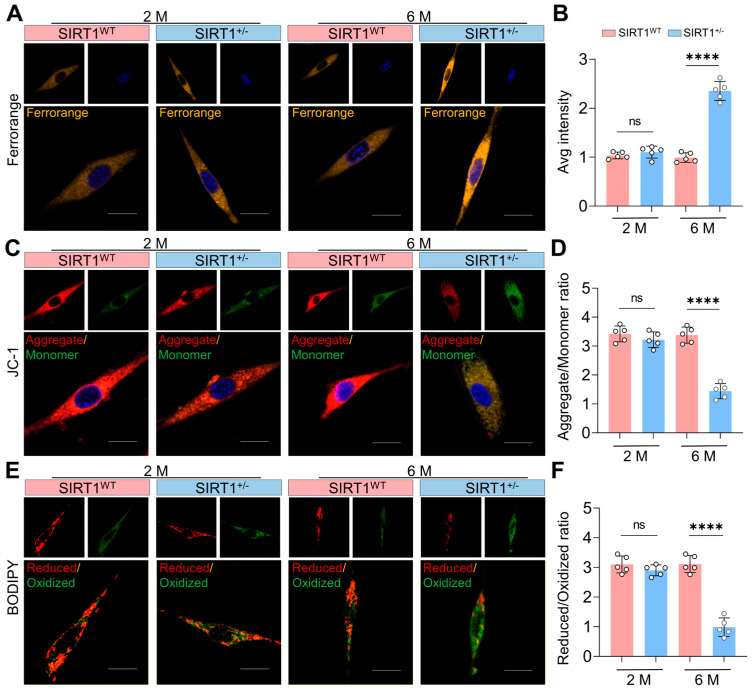
SIRT1 Deficiency Leads to an Increase in Ferroptosis of SSPCs. (**A**,**B**) FerroOrange fluorescence staining (yellow) and quantification of Average intensity in primary SSPCs isolated from the femora of SIRT1^WT^ (red) and SIRT1^+/−^ (blue) mice aged 2 and 6 months. Nuclei were counterstained with Hoechst 33,342 (Blue). Scale bar = 10 μm. *n* = 5. (**C**,**D**) JC-1 assay and quantification of aggregate/monomer ratio in primary SSPCs isolated from the femora of SIRT1^WT^ (red) and SIRT1^+/−^ (blue) mice aged 2 and 6 months. JC-1 monomer was stained green, and JC-1 aggregate was stained red. Nuclei were counterstained with Hoechst 33,342 (Blue). Scale bar = 10 μm. *n* = 5. (**E**,**F**) BODIPY staining and quantification of reduced/oxidized ratio in primary SSPCs isolated from the femora of SIRT1^WT^ (red) and SIRT1^+/−^ (blue) mice aged 2 and 6 months. BODIPY oxidized lipids were stained green, and reduced (non-oxidized) lipids were stained red. Scale bar = 10 μm. *n* = 5. Data are presented as mean ± SD. Each cycle means a sample. Statistical significance was calculated with one-way ANOVA for multiple-group comparisons (**** *p* < 0.0001; ns stands for no significant change).

To delineate the mechanistic basis of SIRT1-mediated osteoporosis, primary SSPCs were isolated from the femora of SIRT1^WT^ and SIRT1^+/−^ mice aged 2 and 6 months for functional assessment of ferroptosis hallmarks. FerroOrange fluorescence staining revealed no significant difference in fluorescence intensity between SSPCs from SIRT1^WT^ and SIRT1^+/−^ mice at 2 months of age. However, by 6 months, SSPCs from SIRT1^+/−^ mice exhibited a robust elevation in FerroOrange signal (Figure 3A,B), indicative of increased labile iron pools, a core initiating event of ferroptosis. JC-1 staining showed comparable MMP between SSPCs from SIRT1^WT^ and SIRT1^+/−^ mice at 2 months. In contrast, SSPCs from SIRT1^+/−^ mice displayed a dramatic reduction in the Aggregate/Monomer ratio at 6 months (Figure 3C,D), consistent with mitochondrial dysfunction, which is a signature feature of ferroptosis. BODIPY staining demonstrated no notable difference in lipid peroxidation levels between the two genotypes at 2 months. However, SSPCs from SIRT1^+/−^ mice exhibited a prominent decrease in the Reduced/Oxidized ratio at 6 months (Figure 3E,F), reflecting marked enhancement of lipid peroxidation, the terminal execution event of ferroptosis.

### 3.4. Identification of HGK as a Potential SIRT1 Activator via Virtual Screening

**Figure 4:**
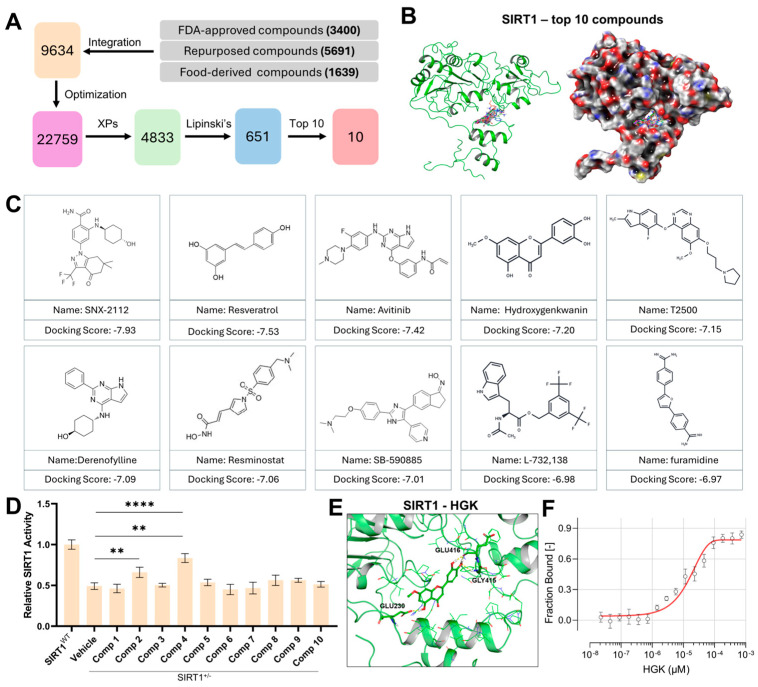
Virtual Screening Identifies Hydroxygenkwanin (HGK) as a Potential SIRT1 Activator. (**A**) Schematic of the virtual screening workflow to identify potential potent SIRT1 activators. (**B**) Schematic diagram illustrating the interaction of the top 10 compounds (multiple color) with SIRT1 protein (green) with the most favorable docking scores. (**C**) The name and chemical formula of the top 10 compounds with the most favorable docking scores with the pocket of SIRT1. (**D**) Quantification of relative SIRT1 activity in primary SSPCs from SIRT1^+/−^ mice treated with 10 compounds. (**E**) 3-D Molecular docking showing the binding scheme of SIRT1 (green) and HGK (multiple color). Hydrogen bonds were shown in yellow. (**F**) Microscale Thermophoresis (MST) analysis of HGK binding to SIRT1 protein. Data are presented as mean ± SD. Statistical significance was calculated with one-way ANOVA for multiple-group comparisons (** *p* < 0.01; **** *p* < 0.0001;). Figure 4C was Created in BioRender. Zhai, Y. (2026) https://BioRender.com/gfur0bd, accessed on 15 March 2026.

We integrated libraries of Repurposing library, Dietary Bioactive Compound Library, and FDA-Approved Compound Library, followed by high-throughput virtual screening targeting the active site of SIRT1 (Figure 4A). A total of 9634 unique compounds were obtained. For ligand preparation, we generated the relevant protonation states and stereoisomers of these compounds, resulting in a final screening library consisting of 22,759 entries. These candidates were further subjected to extra-precision (XP) docking, where 4833 compounds with a docking score < −5 were selected. We then applied Lipinski’s Rule of Five to this subset to assess drug-likeness, ultimately yielding 651 compounds that met the criteria (Figure S1A–E). From these 651 compounds, the top 10 candidates with the most favorable docking scores were chosen for in-depth analysis (Figure 4B). All 10 top-ranked compounds exhibited interactions with the SIRT1 binding pocket harboring the GLU230 residue (Figure 4C). Among these candidates, compound **2** and compound **4** were verified to effectively enhance SIRT1 enzymatic activity, while the effect of compound **4** is better than compound **2** (Figure 4D). Molecular docking analysis revealed that compound **4** (commercially designated as HGK) bound to the GLU230-containing pocket of SIRT1 with a docking score of −7.20 (Figure 4E and Figure S1F). MST test showed that the dissociation constant (Kd) of the SIRT1-HGK complex was 231.93 nM, which exhibited a strong binding profile (Figure 4F).

### 3.5. HGK Inhibits Ferroptosis in SIRT1-Deficient SSPCs

**Figure 5:**
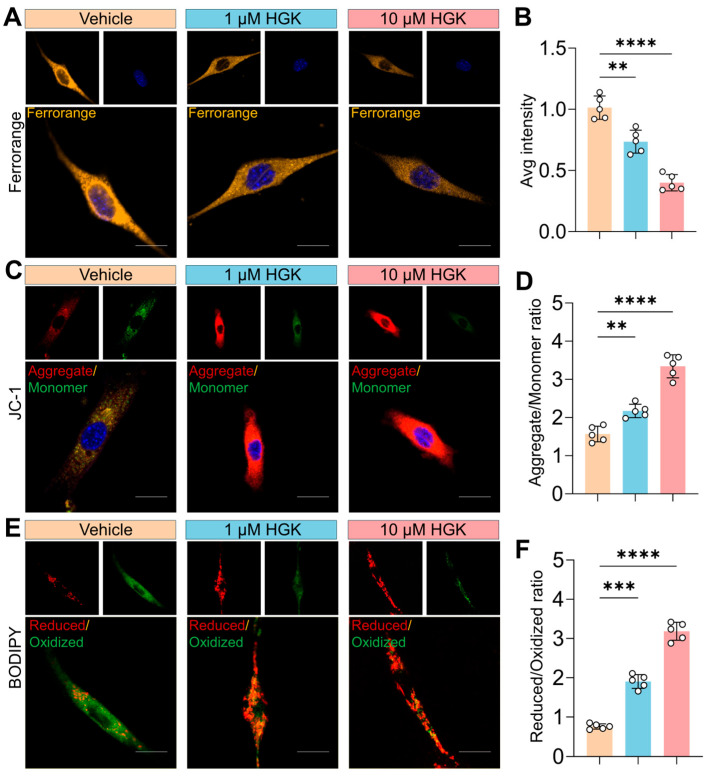
The Ferroptosis in SIRT1-Deficient SSPCs was inhibited by HGK in vitro. (**A**) Representative FerroOrange fluorescence images of SIRT1^+/−^ SSPCs treated with the indicated concentrations of HGK (Vehicle, 1 μM, 10 μM). Nuclei were counterstained with Hoechst 33,342 (Blue). Scale bar = 10 μm. (**B**) Quantification of the average FerroOrange fluorescence (yellow) intensity. *n* = 5. (**C**) Representative JC-1 staining images of SIRT1^+/−^ SSPCs treated with HGK. JC-1 monomers (low MMP) are stained green, and JC-1 aggregates (high MMP) are stained red. Nuclei were counterstained with Hoechst 33,342 (Blue). Scale bar = 10 μm. (**D**) Quantification of the JC-1 Aggregate/Monomer ratio. *n* = 5. (**E**) Representative BODIPY staining images of SIRT1^+/−^ SSPCs treated with HGK. Oxidized lipids are stained green, and reduced lipids are stained red. Scale bar = 10 μm. (**F**) Quantification of the BODIPY Reduced/Oxidized ratio. *n* = 5. Data are presented as mean ± SD. Each cycle means a sample. Statistical significance was calculated with one-way ANOVA for multiple-group comparisons (** *p* < 0.01; *** *p* < 0.001; **** *p* < 0.0001).

To assess whether HGK mitigates ferroptosis in SSPCs from SIRT1^+/−^ mice, we treated these cells with 0 μM (Vehicle), 1 μM, or 10 μM HGK and evaluated core ferroptotic hallmarks. FerroOrange staining revealed that HGK treatment reduced intracellular iron accumulation in SSPCs from SIRT1^+/−^ mice, and the mean fluorescence intensity was significantly lower in the 10 μM HGK group (Figure 5A,B). HGK effectively rescued MMP, with the increased aggregate/monomer ratio being markedly elevated in the 10 μM HGK group compared to untreated SSPCs from SIRT1^+/−^ mice (Figure 5C,D). In addition, 10 μM HGK treatment significantly attenuated lipid peroxidation in SSPCs from SIRT1^+/−^ mice, as evidenced by an increased reduced/oxidized ratio relative to controls (Figure 5E,F). Collectively, these findings demonstrate that HGK suppresses ferroptosis in SIRT1-deficient SSPCs.

### 3.6. HGK Attenuates Osteoporosis in SIRT1-Deficient Mice with a Favorable Biosafety

**Figure 6:**
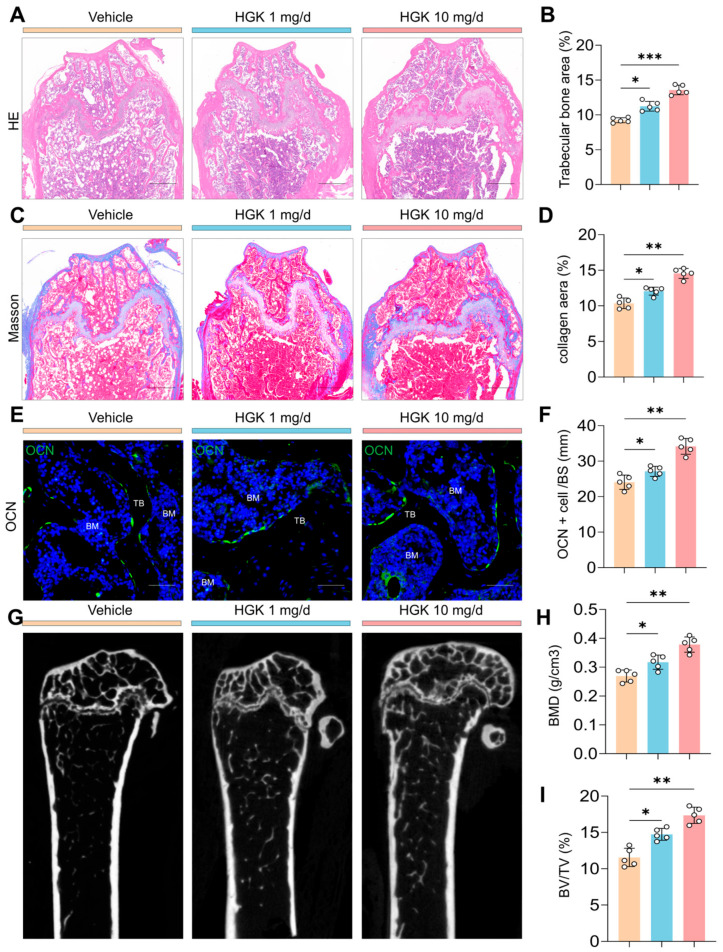
HGK Attenuates Osteoporosis in SIRT1-Deficient Mice. (**A**,**B**) Representative H&E staining images and quantification of femoral trabecular bone of the femoral bone in SIRT1^+/−^ mice with different dosages of HGK. *n* = 5. Scale bar = 400 μm. (**C**,**D**) Representative Masson staining images and quantification of collagen areas of the femoral bone in SIRT1^+/−^ mice with different dosages of HGK. *n* = 5. Scale bar = 400 μm. (**E**,**F**) Immunofluorescence staining images of OCN and quantification in SIRT1^+/−^ mice with different dosages of HGK. *n* = 5. BM indicates bone marrow, TB indicates trabecular bone. Scale bar = 100 μm. (**G**–**I**) Representative coronal section micro-CT images and quantification of the BMD and BV/TV of the femoral bone in SIRT1^+/−^ mice with different dosages of HGK. *n* = 5. Data are presented as mean ± SD. Each cycle means a sample. Statistical significance was calculated with one-way ANOVA for multiple-group comparisons (* *p* < 0.05; ** *p* < 0.01, *** *p* < 0.001).

To assess the therapeutic efficacy of HGK in alleviating premature osteoporosis, we administered HGK (1 mg/d or 10 mg/d) to SIRT1^+/−^ mice for a four-month continuous treatment. H&E staining revealed that 1 mg/d HGK treatment mildly increased trabecular bone area, and 10 mg/d HGK treatment significantly increased trabecular bone area in SIRT1^+/−^ mice (Figure 6A,B). Masson staining showed a marked elevation in collagen area in the 10 mg/d HGK group (Figure 6C,D). Treatment with 10 mg/d HGK significantly increased the number of OCN-positive cells in SIRT1^+/−^ mice (Figure 6E,F). Micro-CT showed a marked elevation in BMD and BV/TV values in the 10 mg/d HGK group (Figure 6G–I). We also evaluated its systemic biosafety in SIRT1^+/−^ mice following administration. No significant alterations were observed in the organ coefficients of major organs between the vehicle and HGK-treated groups (Figure S2A–E). Furthermore, serum biochemical analysis showed that hepatotoxicity markers and hematological profiling remained within the normal range (Figure S2F–L).

### 3.7. RNA-Seq Identifies Core Regulators of HGK in Alleviating Osteoporosis

**Figure 7:**
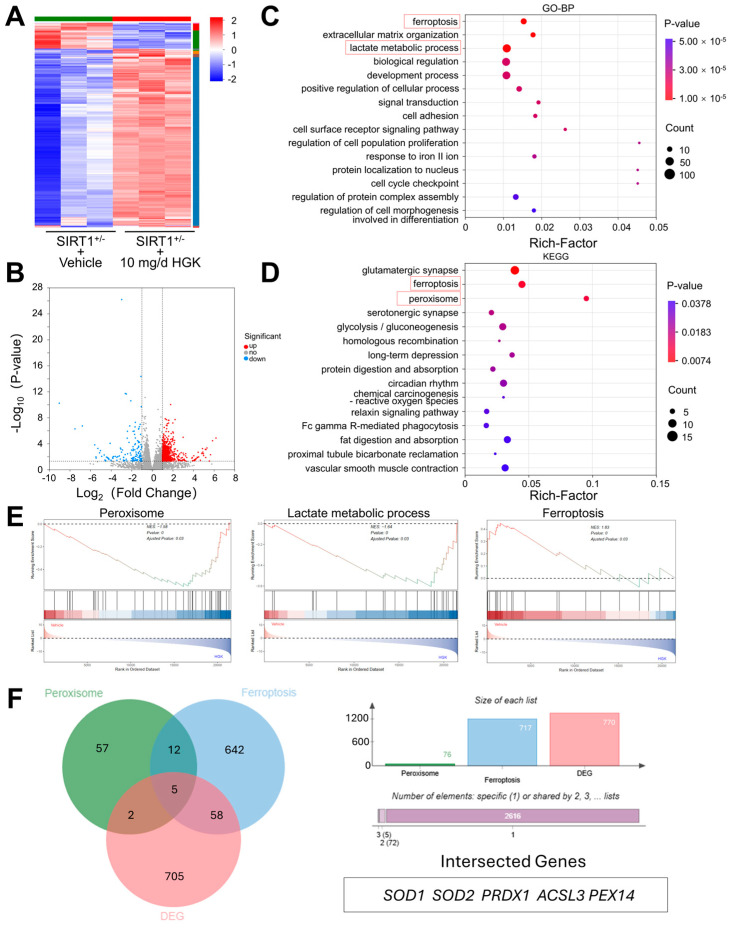
RNA-Seq Identified Core Regulators of HGK in SIRT1 Deficiency-Induced Osteoporosis. (**A**,**B**) Heatmap and Volcano map showing the DEGs of RNA-seq among the SSPCs from SIRT1^+/−^ mice treated with or without HGK. Red means significantly upregulated genes, Blue means significantly downregulated genes. *n* = 3. (**C**,**D**) Gene Ontology Biological process (GO-BP) and KEGG enrichment analysis of the DEGs in SSPCs from SIRT1^+/−^ mice treated with or without HGK. Red boxes mean relevant focused pathways. (**E**) GSEA of the designated GO and KEGG terms among SSPCs from SIRT1^+/−^ mice treated with or without HGK. (**F**) The Venn diagram shows the common intersection of DEGs and the overlapping genes with “ferroptosis” related genes and “peroxisome” related genes.

We performed bulk RNA-seq on SSPCs isolated from vehicle- or 10 mg/d HGK-treated SIRT1^+/−^ Mice. Principal component analysis (PCA) and Pearson correlation coefficient (PCC) demonstrated biological replicate consistency and the robustness of observed transcriptomic changes (Figure S3A,B). We characterized global gene expression patterns via a hierarchical clustering heatmap and a volcano plot (Figure 7A,B). To annotate DEG functional relevance, we performed GO-BP enrichment analysis (Figure 7C), and the most significantly enriched term was “response to oxidative stress” and “lactate metabolic process”. Parallel KEGG pathway enrichment (Figure 7D) identified “ferroptosis” and “peroxisome” as prominent enriched pathways. We next profiled GSEA, and found that genes annotated to “ferroptosis” exhibited downregulation, while “peroxisome” and “lactate metabolic process” pathway genes showed consistent upregulation in SSPCs with HGK treatment (Figure 7E). The DEGs were further intersected with genes from “ferroptosis” related genes and “peroxisome” related genes and yielded 5 overlapping regulators including SOD1, SOD2, PRDX1, ACSL3, and PEX14 (Figure 7F), which represent candidate core factors of HGK in regulating SIRT1-deficiency induced osteoporosis.

### 3.8. HGK Activates SIRT1 and Alleviates Lactylation of SOD1 and PRDX1 in SSPCs

**Figure 8:**
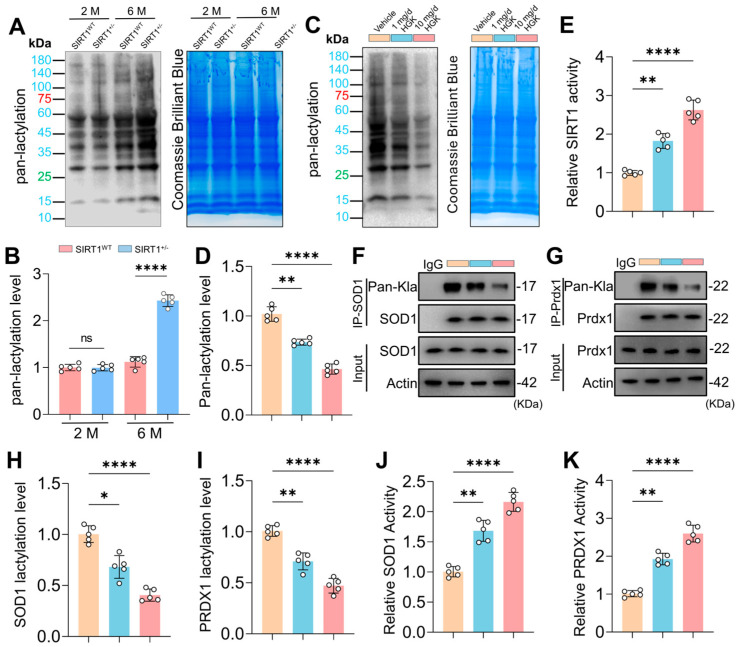
HGK Alleviated Lactylation of Superoxide Dismutase 1 (SOD1) and Peroxiredoxin 1 (PRDX1) and Restored Their Enzymatic Activities. (**A**,**B**) Western blot analysis and quantification of global Pan-Lactylation levels in primary SSPCs isolated from the femora of SIRT1^WT^ and SIRT1^+/−^ mice aged 2 and 6 months. (*n* = 5 individual samples). (**C**,**D**) Western blot analysis and quantification of global Pan-Lactylation levels in primary SSPCs isolated from the femora of SIRT1^+/−^ mice treated with different doses of HGK. (*n* = 5 individual samples). (**E**). Quantification of relative SIRT1 activity in primary SSPCs isolated from the femora of SIRT1^+/−^ mice treated with different doses of HGK. (*n* = 5 individual samples). (**F**–**I**) IP analysis and quantification to detect the lactylation levels of SOD1 and PRDX1 in primary SSPCs isolated from the femora of SIRT1^+/−^ mice treated with different doses of HGK. (*n* = 5 individual samples). (**J**,**K**) Quantification of SOD1 and PRDX1 activities in primary SSPCs isolated from the femora of SIRT1^+/−^ mice treated with different doses of HGK. (*n* = 5 individual samples). Data are presented as mean ± SD. Each cycle means a sample. Statistical significance was calculated with one-way ANOVA for multiple-group comparisons (* *p* < 0.05; ** *p* < 0.01; **** *p* < 0.0001; ns stands for no significant change).

SIRT1 is reported to be a potential delactylase, we then explored whether HGK alleviated SSPCs ferroptosis by activating SIRT1 and regulating protein lactylation. We observed a marked upregulation of Pan-Lactylation levels in SSPCs from SIRT1^+/−^ mice, which was most pronounced at 6 months (Figure 8A,B), indicating widespread enhancement of protein lactylation driven by SIRT1 deficiency. In comparison, we found that the Pan-Lactylation levels in SSPCs from SIRT1^+/−^ mice were reduced after HGK treatment (Figure 8C,D). The SIRT1 activity was elevated in SSPCs from SIRT1^+/−^ mice receiving HGK treatment (Figure 8E). In addition, IP experiments showed a decrease in lactylation levels of SOD1 and PRDX1 in SSPCs from SIRT1^+/−^ mice receiving HGK treatment (Figure 8F–I). For the remaining candidate proteins, SOD2, ACSL3, and PEX14, IP analyses revealed no significant lactylation alterations (Figure S4A–C). We also found that the upregulated levels of lactylated SOD1 and PRDX1 in SSPCs from SIRT1^+/−^ mice were accompanied by reduced enzymatic activities of SOD1 and PRDX1 (Figure S5A,B). In comparison, the enzymatic activities of SOD1 and PRDX1 were restored in SSPCs from SIRT1^+/−^ mice receiving HGK treatment (Figure 8J,K). Collectively, these data demonstrated that HGK can activate the delactylase capacity of SIRT1 to mediate the de-lactylation of SOD1 and PRDX1, which may elevate the suppressed enzymatic activity of SOD1 and PRDX1 by lactylation.

## 4. Discussion

In this study, we employed large-cohort RNA-seq data mining to identify that reduced SIRT1 expression in SSPCs correlated with the progression of aging-related osteoporosis. Subsequently, by generating SIRT1^+/−^ mice, we demonstrated that SIRT1 deficiency induces premature osteoporosis, which was accompanied by elevated ferroptosis in SSPCs. To identify therapeutic agents, we integrated libraries of repurposed compounds, dietary bioactive compounds, and FDA-approved compounds and screened for molecules targeting the active site of SIRT1. This effort identified HGK as a potent SIRT1 activator that directly binds and activates SIRT1. Notably, administration of HGK to SIRT1^+/−^ mice effectively alleviated osteoporosis-related phenotypes with favorable biosafety profiles. Mechanistic assays then showed that HGK reduced global pan-lactylation in SSPCs, alongside decreased lactylation level of the SOD1 and PRDX1 proteins. Collectively, this study uncovered HGK as a novel and potent SIRT1 activator in treating osteoporosis.

Osteoporosis is a senescence-associated orthopedic disorder characterized by complex and heterogeneous pathogenic mechanisms [45]. Recent advances in transcriptomics technologies have positioned high-throughput RNA-seq data mining from public repositories as a core driver for target discovery in orthopedic disorders. Numerous studies have integrated gene expression profiles of pathological tissues (e.g., from osteoporosis, osteoarthritis, intervertebral disk degeneration, and osteosarcoma) [46,47,48], identifying key DEGs and hub genes closely linked to pathogenic processes, including inflammatory microenvironment dysregulation, extracellular matrix remodeling, and oxidative stress. This translational strategy, which bridges big data to precision targets, not only markedly shortens the timeline for traditional therapeutic target discovery but also elucidates complex molecular regulatory networks from a systems biology standpoint, thereby laying a robust theoretical foundation for deciphering the molecular mechanisms of orthopedic disorders and developing novel therapeutic strategies [49,50]. To explore age-related drivers of osteoporosis, we analyzed RNA-seq datasets from 288 SSPCs samples isolated from young mice and 288 SSPCs samples from aged mice. This analysis identified 225 upregulated and 208 downregulated genes. Notably, these DEGs were significantly enriched for the “ferroptosis” term, suggesting a tight association between these cellular alterations and osteoporosis pathogenesis.

Among these DEGs, we identified that multiple members of the SIRT protein family, including SIRT1, SIRT2, SIRT3, and SIRT6, were downregulated in SSPCs isolated from aged mice. Notably, SIRT1 exhibited the most prominent reduction in expression among these SIRT family members. SIRT1 plays a central protective role in skeletal disorders by establishing a multidimensional defense network that enhances cellular detoxification capacity, directly alleviates oxidative stress, and promotes mitochondrial biogenesis and functional maintenance [51,52]. This network not only counteracts pathological factors leading to bone damage, but also potentially intervenes in downstream pathways closely related to bone injury, including cellular senescence and ferroptosis [53,54,55]. Given that global SIRT1 knockout leads to embryonic lethality, we generated SIRT1 ^+/−^ heterozygous mice. Regarding the use of 2-month-old and 6-month-old mice, these animals were selected because they represent young adult mice and yield a relatively stable population of bone-derived SSPCs, while minimizing the confounding effects of aging on SSPC stemness and ferroptosis-related responses. In addition, SSPCs isolated from mice at these ages generally maintain relatively strong osteogenic potential. Phenotypic analysis revealed that these SIRT1 ^+/−^ mice did not exhibit obvious osteoporotic features at 2 months of age. However, they developed a prominent premature osteoporotic phenotype by 6 months, accompanied by marked ferroptosis in SSPCs. This finding is consistent with previous reports demonstrating that SIRT1 deficiency induces ferroptosis in models of Parkinson’s disease [56] and hepatic steatosis disease [57].

SIRT1 has been reported to have multiple activators, including natural products (e.g., resveratrol, quercetin, curcumin) [58,59,60], synthetic small molecules (e.g., SRT1720, SRT2104, SRT1460) [61,62,63], and NAD+ precursors (e.g., NMN, NR) [64,65]. However, previous studies have reported that natural products like resveratrol exhibit poor bioavailability, synthetic small molecules are associated with high synthesis costs and inconsistent clinical trial outcomes, and NAD+ precursors are costly and prone to off-target effects [66,67,68]. CADD is an emerging and rapidly advancing approach for drug screening. Leveraging high-throughput in silico virtual screening, this technology circumvents the high cost and long cycle of traditional trial-and-error screening, enabling rapid identification of bioactive compounds with optimized molecular interactions for subsequent biological validation [69,70]. It is particularly well-suited for drug repurposing, exploring novel therapeutic effects of known compounds [71]. We therefore integrated libraries of repurposed compounds, dietary bioactive compounds, and FDA-approved compounds, followed by high-throughput virtual screening targeting the active site of SIRT1 harboring the GLU230 residue [72]. Among the 9634 compounds screened, HGK exhibited the most potent SIRT1-activating activity, which is better than resveratrol. HGK, a flavonoid analog with favorable lipid solubility, has been shown to exert anti-oxidative stress and anti-apoptotic effects in various disease models [73]; however, its regulatory role in SIRT1 and therapeutic potential in osteoporosis remain unreported. In vivo animal experiments demonstrated that HGK administration effectively restored SIRT1 activity and alleviated osteoporosis. Furthermore, hematological analyses and visceral organ/body weight ratio assessments confirmed a favorable biosafety profile of HGK, highlighting its potential for clinical translation.

We therefore sought to delineate the underlying mechanism by which HGK suppressed ferroptosis in SSPCs and alleviated osteoporosis. RNA-seq analysis revealed that DEGs were significantly enriched in pathways including peroxisome function, oxidative stress, ferroptosis, and lactate metabolism. To identify core regulatory factors, we intersected DEGs with genes annotated to the peroxisome and ferroptosis pathways, yielding five key candidates: SOD1, SOD2, PEX14, ACSL4, and PRDX1. Notably, a 2019 study identified that lactate can donate lactyl groups to lysine residues of proteins, thereby altering protein function and regulating downstream cellular events, and this novel post-translational modification was termed lactylation [74]. Accumulating evidence has established SIRT1 as a canonical eraser of protein lactylation [75], with reports demonstrating its role in regulating lactylation processes of IRF9 [76], as well as its involvement in the pathogenesis of sepsis-induced ferroptosis in myocardial injury [77]. Analysis of global pan-lactylation levels demonstrated that SIRT1 deficiency elevated pan-lactylation in SSPCs, while HGK treatment significantly reduced pan-lactylation in SSPCs. Subsequent IP-coupled Western blotting assays confirmed that HGK reduced the lactylation levels of SOD1 and PRDX1. SIRT1 deficiency resulted in the decline of the enzymatic activities of both SOD1 and PRDX1 in SSPCs, while we observed a significant elevation in the enzymatic activities of both SOD1 and PRDX1 after HGK treatment, concomitantly with decreased lactylation in SOD1 and PRDX1. Given that SOD1 and PRDX1 are well-characterized key anti-ferroptotic proteins [78,79], we hypothesize that HGK activates SIRT1 to reduce the lactylation of SOD1 and PRDX1, which restores their anti-ferroptotic enzymatic activities, thereby alleviating ferroptosis in SSPCs and ameliorating osteoporosis.

This study has several limitations. First, HGK and its closely related analog genkwanin are both flavonoids that have been reported to exert protective effects against oxidative stress. Genkwanin has been reported to activate SIRT1 in colitis, acute lung injury, and skin flap survival [80,81,82]. In the present study, we provide the first evidence that hydroxygenkwanin exhibits potent SIRT1-activating activity in our experimental system, thereby attenuating SSPC injury and alleviating osteoporosis. Future studies will further compare the anti-osteoporotic effects of HGK and genkwanin. Second, As a natural compound derived from Daphne genkwa, HGK has been detected in plasma in previous pharmacokinetic studies following oral administration of Daphne genkwa extract. However, direct data on the absolute oral bioavailability of purified HGK are still lacking. HGK has been shown to undergo glucuronidation in human liver microsomes, suggesting that its oral exposure may be substantially influenced by host metabolism [83]. As for gut microbiota-mediated metabolism, HGK-specific direct evidence is currently unavailable; nevertheless, given that flavonoids are commonly subject to gut microbial biotransformation, the possibility that oral exposure to HGK is also affected by this process cannot be excluded [84]. Future studies are therefore warranted to further define the oral bioavailability of HGK and to compare the therapeutic effects of oral versus non-oral administration.

## 5. Conclusions

Our study establishes SIRT1 as a potential therapeutic target for aging-related osteoporosis. Computational high-throughput virtual screening led to the identification of HGK as a novel SIRT1 activator. Oral administration of HGK effectively attenuates osteoporosis induced by SIRT1 deficiency, while exhibiting a favorable biosafety profile. HGK activates SIRT1 to reduce the lactylation of SOD1 and PRDX1, which may restore their anti-ferroptotic enzymatic activities, thereby alleviating ferroptosis in SSPCs and ameliorating osteoporosis. HGK possesses certain potential as a promising translational candidate for the treatment of osteoporosis in the future.

## Data Availability

Data from RNA-seq analysis (Section 2.1) were obtained from the Gene Expression Omnibus (GEO) database under accession number GSE161946. The dataset is publicly available. The RNA-sequencing data from Section 2.12 have been deposited in the NCBI BioProject database under accession number PRJNA1464077 and are publicly accessible at: https://www.ncbi.nlm.nih.gov/bioproject/PRJNA1464077.

## Supplementary Materials

The following supporting information can be downloaded at: https://www.mdpi.com/article/10.3390/antiox15050612/s1, Figure S1: Drug-likeness evaluation of candidate compounds based on Lipinski’s Rule of Five. The physicochemical properties of the candidate compounds were analyzed to assess their drug-likeness. The scatter plots illustrate the distribution of compounds based on five key criteria (A) Molecular Weight (MW), (B) LogP (partition coefficient), (C) Hydrogen Bond Donors (HBD), (D) Hydrogen Bond Acceptors (HBA), and (E) Rotatable Bonds (RB). The light blue shaded areas represent the optimal range for each parameter according to drug-likeness rules. Compounds falling within these regions were selected for further screening. (F) 2D schematic molecular docking results of HGK with SIRT1; Figure S2: Evaluation of HGK biosafety in vivo. (A–E) Organ coefficients (organ weight/body weight × 100%) of the liver, heart, spleen, lung, and kidneys in SIRT1^+/−^ mice treated with Vehicle, 1 mg/d HGK, or 10 mg/d HGK. (F,G) Serum levels of liver function markers ALT and AST in SIRT1^+/−^ mice treated with Vehicle, 1 mg/d HGK, or 10 mg/d HGK. (H–L) Hematological parameters, including WBC, RBC, HGB, MCH, and PLT in SIRT1^+/−^ mice treated with Vehicle, 1 mg/d HGK, or 10 mg/d HGK. Data are presented as mean ± SD. Each cycle means a sample. Statistical significance was determined by one-way ANOVA (ns stands for no significant change); Figure S3: Validation of RNA-seq data quality and consistency. (A) Principal Component Analysis (PCA) plot showing the distinct separation between the transcriptomic profiles of the SSPCs from SIRT1^+/−^ mice treated with or without HGK. The percentage of variance explained by PC1 and PC2 is indicated on the axes. (B) Pear-son Correlation Coefficient (PCC) heatmap illustrating the high correlation between biological replicates within each group. The color scale (red to blue) represents the correlation coefficient value, with red indicating high correlation (close to 1.0); Figure S4: Validation of HGK-mediated delactylation effects. (A) IP analysis to detect the lactylation levels of PEX14, ACSL3, and SOD2 in primary SSPCs isolated from the femora of SIRT1^+/−^ mice treated with different dose of HGK. (D,E) Quantifications to detect the lactylation levels of PEX14, ACSL3, and SOD2 in primary SSPCs isolated from the fem-ora of SIRT1^+/−^ mice treated with different dose of HGK (*n* = 5 individual samples). Data are presented as mean ± SD. Each cycle means a sample. Statistical significance was calculated with one-way ANOVA for multiple-group com-parisons (ns stands for no significant change); Figure S5: Validation of SOD1 and PRDX1 activity in SIRT1-deficient mice. (A,B). Quantification of SOD1 and PRDX1 activities in primary SSPCs isolated from the femora of SIRT1WT and SIRT1^+/−^ mice. *n* = 5. Data are presented as mean ± SD. Each cycle means a sample. Statistical significance was calculated with one-way ANOVA for multiple-group comparisons (**** *p* < 0.0001; ns stands for no significant change).

## Abbreviations

The following abbreviations are used in this manuscript:

SIRT1	Sirtuin 1
SSPCs	Skeletal Stem/Progenitor Cells
HGK	Hydroxygenkwanin
RNA-seq	RNA-sequencing
CADD	Computer-Aided Drug Design
SOD1	Superoxide Dismutase 1
PRDX1	Peroxiredoxin 1
BMSCs	Bone Marrow Mesenchymal Stem Cells
DEGs	Differentially Expressed Genes
GSEA	Gene Set Enrichment Analysis
GO	Gene Ontology
KEGG	Kyoto Encyclopedia of Genes and Genomes
BMD	Bone Mineral Density
BV/TV	Bone Volume/Total Volume
OCN	Osteocalcin
ROI	Region of Interest
MMP	Mitochondrial Membrane Potential
MST	Microscale Thermophoresis
WT	Wild-type
VOI	Volume of Interest
