# Supplementary material for: A Novel SIRT1 Activator Hydroxygenkwanin Alleviates Osteoporosis by Inhibiting Ferroptosis and Lactylation in Skeletal Stem/Progenitor Cells
Source: Antioxidants (Basel). 2026 May 12;15(5):612. doi: 10.3390/antiox15050612 (PMC13203516; doi:10.3390/antiox15050612)
Supplement: Supplementary file 1 [file antioxidants-15-00612-s001.zip › antioxidants-4220944-supplementary.pdf]

Supplementary Materials

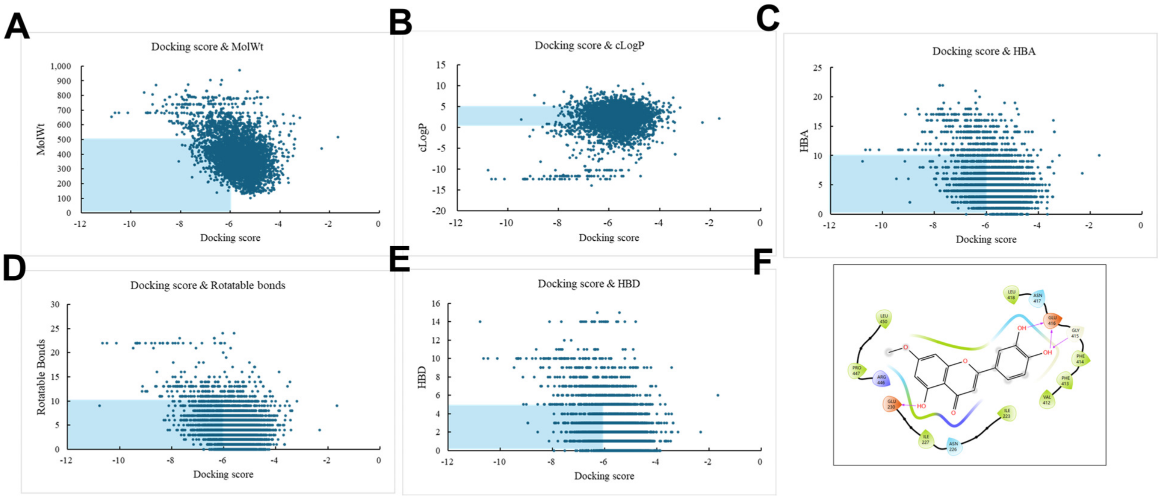

Figure S1. Drug-likeness evaluation of candidate compounds based on Lipinski's Rule of Five. The physicochemical properties of the candidate compounds were analyzed to assess their drug-likeness. The scatter plots illustrate the distribution of compounds based on five key criteria (A) Molecular Weight (MW), (B) LogP (partition coefficient), (C) Hydrogen Bond Donors (HBD), (D) Hydrogen Bond Acceptors (HBA), and (E) Rotatable Bonds (RB). The light blue shaded areas represent the optimal range for each parameter according to drug-likeness rules. Compounds falling within these regions were selected for further screening. (F) 2D schematic molecular docking results of HGK with SIRT1.

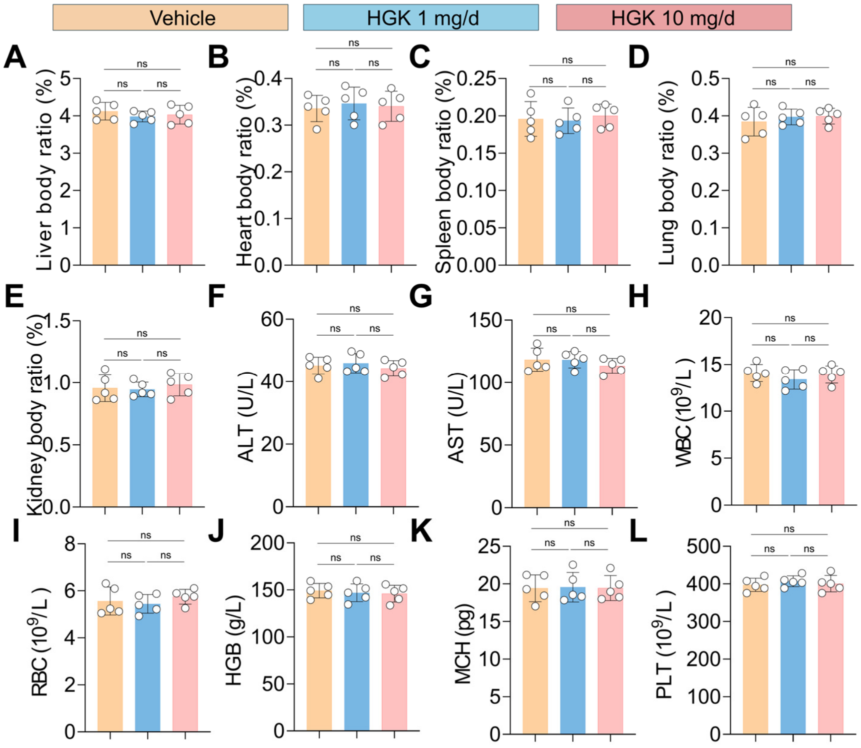

Figure S2. Evaluation of HGK biosafety *in vivo*. (A–E) Organ coefficients (organ weight/body weight × 100%) of the liver, heart, spleen, lung, and kidneys in SIRT1+/- mice treated with Vehicle, 1 mg/d HGK, or 10 mg/d HGK. (F–G) Serum levels of liver function markers ALT and AST in SIRT1+/- mice treated with Vehicle, 1 mg/d HGK, or 10 mg/d HGK. (H–L) Hematological parameters, including WBC, RBC, HGB, MCH, and PLT in SIRT1+/- mice treated with Vehicle, 1 mg/d HGK, or 10 mg/d HGK. Data are presented as mean ± SD. Each cycle means a sample. Statistical significance was determined by one-way ANOVA (ns stands for no significant change).

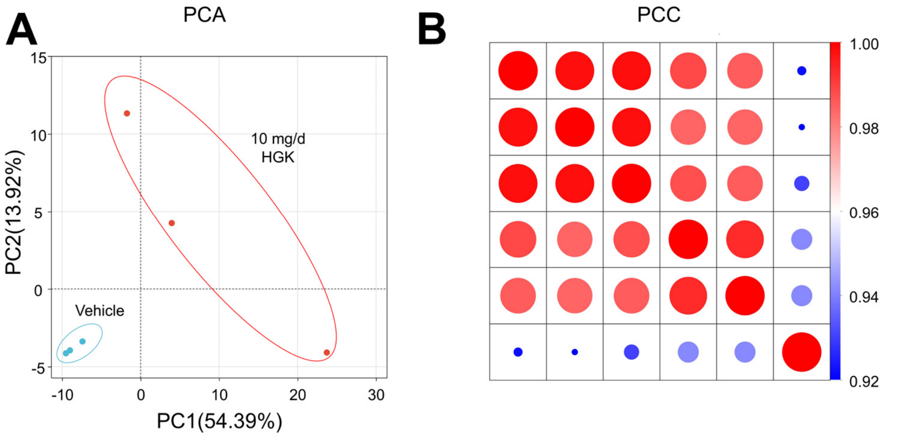

Figure S3. Validation of RNA-seq data quality and consistency. (A) Principal Component Analysis (PCA) plot showing the distinct separation between the transcriptomic profiles of the SSPCs from SIRT1$^{+/-}$ mice treated with or without HGK. The percentage of variance explained by PC1 and PC2 is indicated on the axes. (B) Pearson Correlation Coefficient (PCC) heatmap illustrating the high correlation between biological replicates within each group. The color scale (red to blue) represents the correlation coefficient value, with red indicating high correlation (close to 1.0).

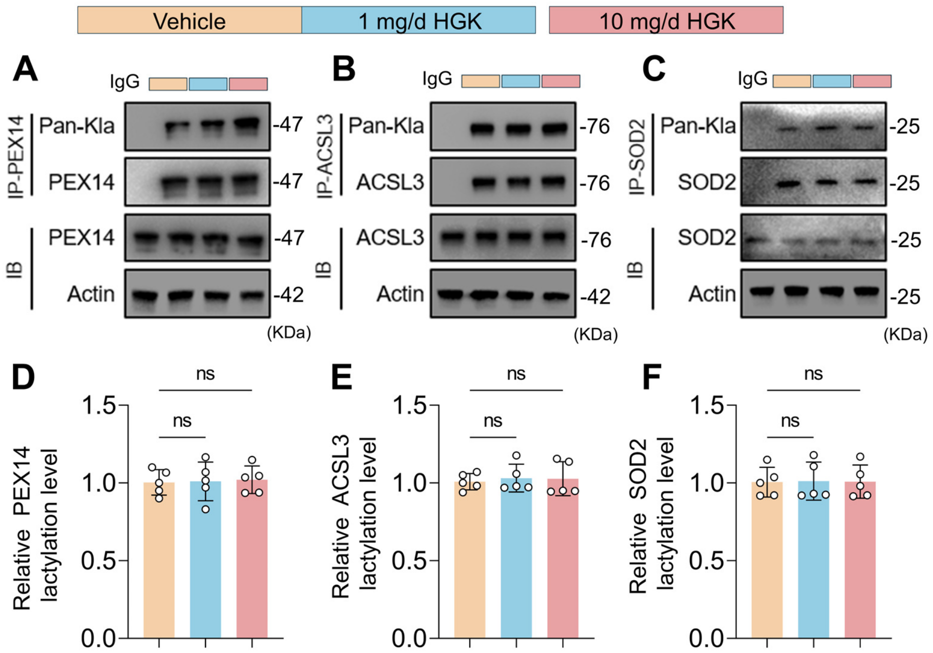

Figure S4. Validation of HGK-mediated delactylation effects. (A) IP analysis to detect the lactylation levels of PEX14, ACSL3, and SOD2 in primary SSPCs isolated from the femora of SIRT1$^{+/-}$ mice treated with different dose of HGK. (D-E) Quantifications to detect the lactylation levels of PEX14, ACSL3, and SOD2 in primary SSPCs isolated from the femora of SIRT1$^{+/-}$ mice treated with different dose of HGK (n = 5 individual samples). Data are presented as mean ± SD. Each cycle means a sample. Statistical significance was calculated with one-way ANOVA for multiple-group comparisons (ns stands for no significant change).

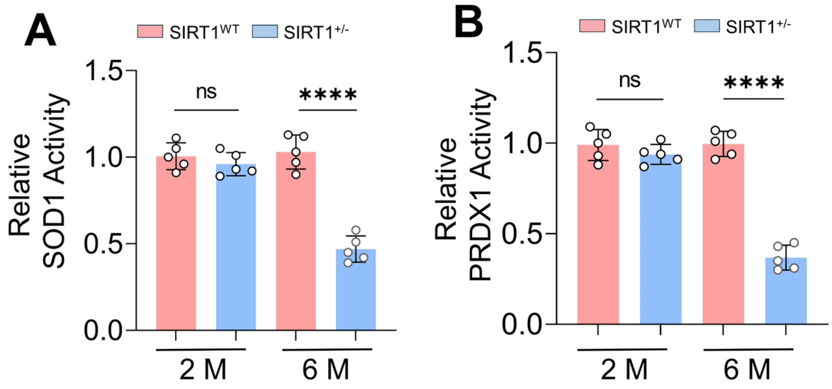

Figure S5. Validation of SOD1 and PRDX1 activity in SIRT1-deficient mice. (A-B). Quantification of SOD1 and PRDX1 activities in primary SSPCs isolated from the femora of $SIRT1^{WT}$ and $SIRT1^{+/-}$ mice. n = 5. Data are presented as mean ± SD. Each cycle means a sample. Statistical significance was calculated with one-way ANOVA for multiple-group comparisons (**** $p < 0.0001$; ns stands for no significant change).
